# Curcumin protects against hepatic ischemia/reperfusion induced injury through inhibiting TLR4/NF-κB pathway

**DOI:** 10.18632/oncotarget.18676

**Published:** 2017-06-27

**Authors:** Lu Wang, Ning Li, Dongdong Lin, Yunjin Zang

**Affiliations:** ^1^ Department of Surgery and Liver Transplantation Center, Capital Medical University Affiliated Youan Hospital, Beijing, China

**Keywords:** hepatic ischemia reperfusion injury, curcumin, TLR4, NF-κB, inflammatory cytokine

## Abstract

The TLR4/NF-κB pathway had important roles in hepatic ischemia/reperfusion (I/R) injury. In this study, we reported a protective effect of curcumin against hepatic I/R injury via TLR4/NF-κB pathway. Curcumin significantly inhibited cell apoptosis, and decreased levels of LDH and production of TNF-a, IL-1b, and IL-6 in the cell supernatant. In addition, curcumin ameliorated elevated TLR4 and NF-κB caused by hypoxia/reoxygenation stimulation in BRL-3A cells. In vivo assays revealed that curcumin reduce levels of ALT and AST, and reversed TLR4/NF-κB signaling pathway caused by hepatic I/R stimulation in liver tissues. These results suggested that curcumin ameliorates hepatic I/R injury, which may be mediated in part via the TLR4/NF-κB signaling pathway.

## INTRODUCTION

Hepatic ischemia-reperfusion (I/R) injury plays an important role in liver transplantation and resection surgery [1–2]. Patients who suffer from hepatic I/R are exposed to enormous pain. Excessive inflammation plays a predominant role in the initiation and progression of hepatic I/R injury [3]. Recent results have demonstrated that Toll-like receptor 4 (TLR4)/nuclear factor (NF)-kB pathway could induce inflammatory response and then regulate the hepatic I/R injury [4–5]. TLR pathway could cause systematic inflammation by directly or indirectly activating NF-κB pathway [6–7]. Therefore, it is urgent to develop new and effective therapies for the treatment of hepatic I/R injury.

Curcumin, a natural polyphenol found in the roots of *Curcuma longa*, is widely used in Ayurvedic medicine for centuries [8–9]. Although some studies have reported that curcumin can modulate kidney and myocardial I/R injury [10–11], however, the mechanisms by which curcumin regulates hepatic I/R injury remains unknown. In this study, we investigate whether curcumin administration has a protective effect on hepatic I/R injury associated with inhibition of TLR4/NF-kB pathway. Our results have demonstrated that curcumin alleviates hepatic I/R injury by inhibition of TLR4/NF-kB pathway.

## RESULTS

### Curcumin alleviated cell damage

**Figure 1:**
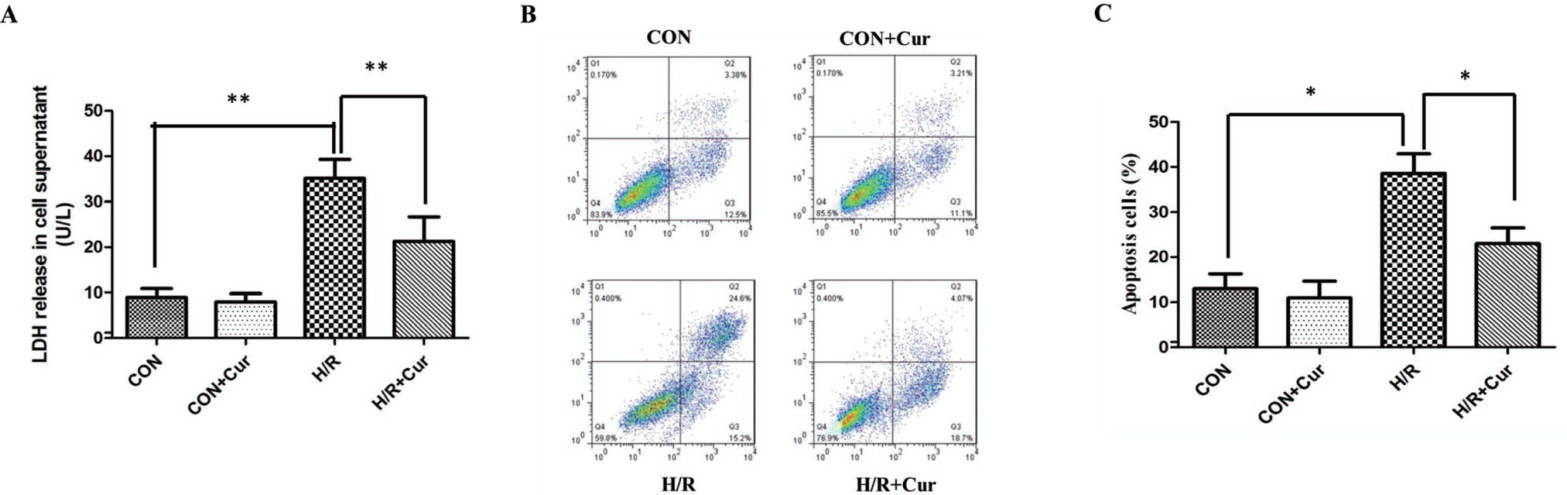
(**A**) Effects of curcumin (10 μM) on LDH release in BRL-3A cells subjected to hypoxia/reoxygenation; (**B**) Effects of curcumin on apoptosis of BRL-3A cells subjected to hypoxia/reoxygenation; (**C**) Apoptosis data are expressed as mean ± standard deviation (SD, *n* = 3); *P < 0.05, **P < 0.01.

The levels of LDH was significantly increased in the H/R group when compared with the CON group (35.13 ± 4.17 vs 8.93 ± 1.94 U/L; Figure 1A; *P* < 0.01). Besides, the levels of LDH did not significantly change in the CON+Cur group. Interestingly, curcumin (10 μM) inhibited the release of LDH in the H/R+Cur group (21.25 ± 5.38 U/L, *P* < 0.01). In addition, H/R stimulation dramatically increased the apoptosis rates of BRL-3A cells (38.63 ± 4.31%, *P* < 0.05), when compared with the CON group. However, the difference of apoptosis rates in the CON and CON+Cur group was not significanct (Figure 1B and 1C; *P* = 0.109). Moreover, treatment with curcumin could significantly reduce the apoptosis rates in the H/R+Cur group (23.00 ± 3.46; *P* < 0.05).

### Curcumin inhibited of TLR4/NF-κB pathway

**Figure 2:**
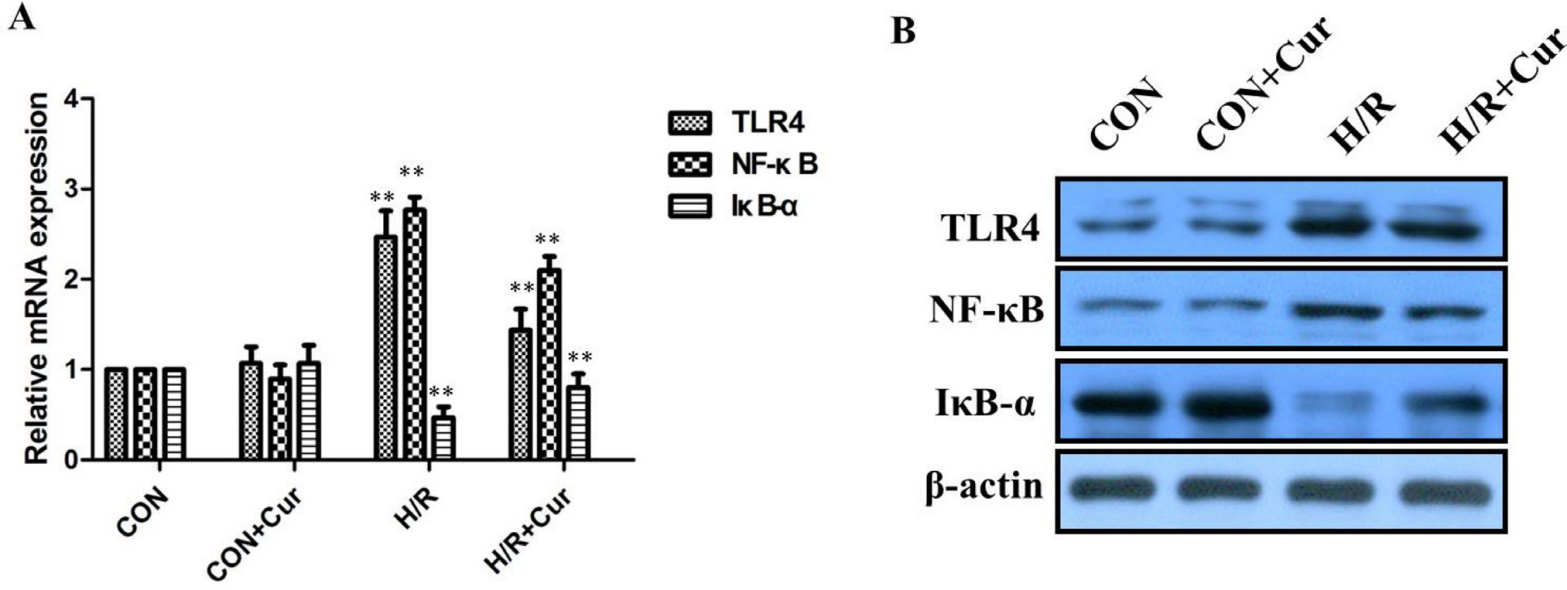
(**A**) Changes in TLR4, NF-κB, and IκB-α mRNA and proteins expression *in vitro*. A: qRT-PCR analysis of TLR4, IκB-α, and NF-κB mRNA expression in BRL-3A cells; (**B**) Western blot analysis of TLR4, IκB-α, and NF-κB protein expression in BRL-3A cells; **P < 0.01.

Our results revealed a dramatic increase of TLR4 and NF-κB expression in the H/R group, whereas IκB-α was significantly decreased compared with control. In contrast, curcumin (10 μM) could significantly reduce the TLR4 and NF-κB levels, while the IκB-α was obviously increased by curcumin compared with the H/R group (Figure 2A, 2B; *P* < 0.01). The expressions of TLR4, NF-κB, and IκB-α between the CON and CON+Cur groups was not significantly change.

### Curcumin suppressed inflammatory cytokines production

**Figure 3:**
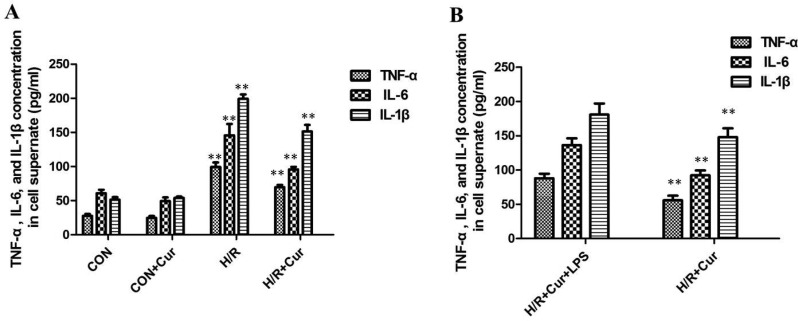
(**A**) Effects of curcumin on TNF-α, IL-6, and IL-1β production in BRL-3A cells, **P < 0.01; (**B**) levels of TNF-α, IL-6, and IL-1β in the culture medium with LPS stimulation were significantly increased than cells treated with curcumin alone, **P < 0.01.

To further investigate the effect of curcumin on the levels of pro-inflammatory cytokines, we analyzed the concentration of TNF-α, IL-6, and IL-1β in the culture medium by ELISA. The levels of TNF-α, IL-6, and IL-1β of BRL-3A cells subjected to H/R in the culture medium were found to be significantly augmented compared to the CON group (*P* < 0.01). The increase of TNF-α, IL-6, and IL-1β levels could be effectively suppressed by curcumin (Figure 3A, *P* < 0.01)

To evaluate the influence of TLR4 activation on curcumin, BRL-3A cells were pretreated with curcumin and then stimulated with LPS or DMSO alone for another 4 h. Expression of the inflammatory cytokines TNF-α, IL-6, and IL-1β were then measured by ELISA as a readout of TLR4 activation. This experiment revealed that levels of TNF-α, IL-6, and IL-1β in the culture medium with LPS stimulation were significantly increased than cells treated with curcumin alone (Figure 3B; *P* < 0.01).

### Curcumin decreased levels of ALT, AST, and inflammatory cytokines in hepatic I/R-injured rats

**Figure 4:**
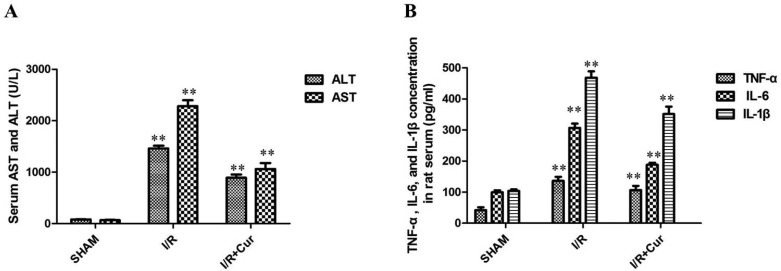
(**A**) Curcumin obviously reduced the levels of ALT and AST in rat serum; (**B**) Effects of curcumin on TNF-α, IL-6, and IL-1β production in rat serum; **P < 0.01.

To determine the effects of curcumin on, they were pretreated with either saline or curcumin and subjected to partial liver warm ischemia. The level of ALT and AST were increased remarkably in the 24 hours after reperfusion of hepatic I/R-injured rat. In contrast, 100 mg/kg/day of curcumin could significantly reduce the levels of ALT and AST in rat serum (Figure 4A; *P* < 0.01). We next examined inflammatory response in rats after hepatic I/R injury by detecting the production of inflammatory cytokines. The results showed that the TNF-α, IL-6, and IL-1β levels of rat serum were significantly higher in hepatic I/R group than SHAM group. However, the serum levels of TNF-α, IL-6, and IL-1β were also suppressed by curcumin treatment (100 mg/kg/day; Figure 4B; *P* < 0.01).

### Curcumin inhibited TLR4/NF-kB Pathway in hepatic I/R-injured rats

**Figure 5:**
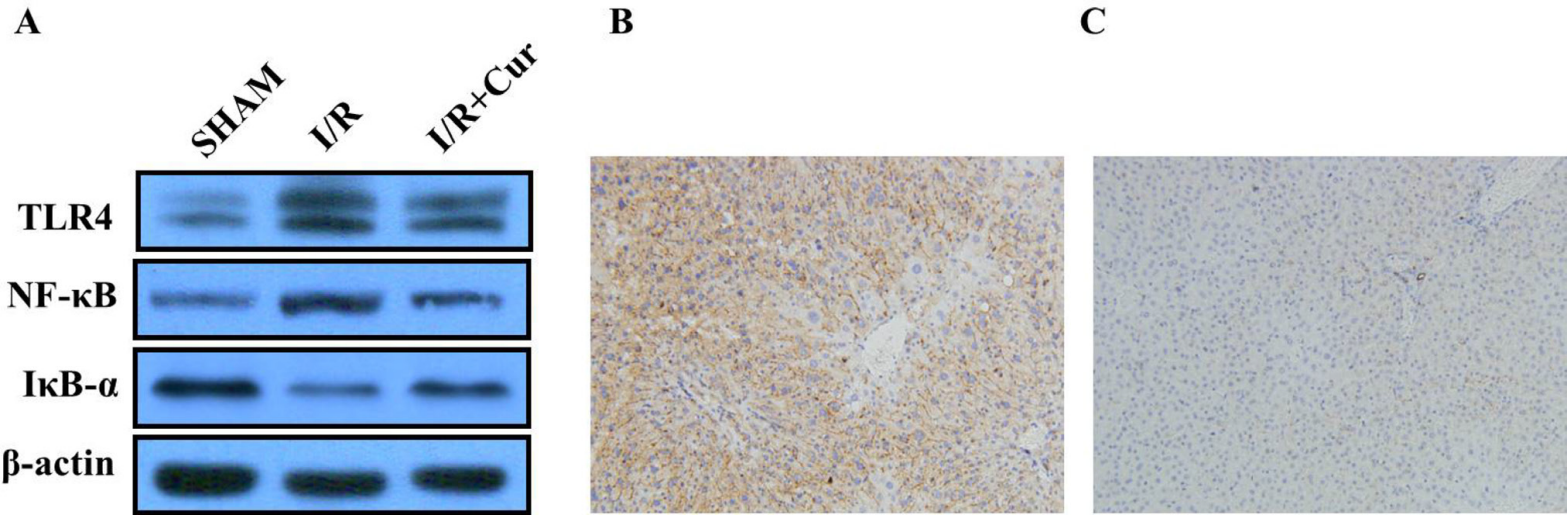
(**A**) Western blot analysis of TLR4, IκB-α, and NF-κB protein expression in rat liver tissues; (**B**) Positive expression of TLR4 in rat liver tissues; (**C**) Negative expression of TLR4 in rat liver tissues.

We then examined the effect of curcumin on the TLR4/NF-kB signaling pathway in ischemic liver tissues. The expression of TLR4 and NF-κB were significantly increased in the hepatic I/R group, however, the expressions of TLR4 and NF-κB were decresed after the treatment of curcumin (Figure 5A), indicating that the anti-inflammatory effect of curcumin is mediated by inhibition of TLR4/NF-κB pathway in hepatic I/R-injured rats. The IHC analysis showed the positive expression of TLR4 in the hepatic I/R group. However, treatment with curcumin reduced the expressions of TLR4 (Figure 5B).

## DISCUSSION

I/R injury is the main cause of early allograft dysfunction after organ transplantation. In recent years, the number of patients at risk of hepatic I/R injury is increasing. Research aimed at the prevention and treatment of hepatic I/R is urgent. Curcumin is a yellow colored polyphenolic compound derived from Curcuma longa. More and more research proves that curcumin could exert a variety of pharmacological and biological properties [12–13]. There have been three articles reporting of the effects of curcumin on kidney and myocardial I/R injury, however, the impact of curcumin on hepatic I/R has not been investigated. The aim of the present study was to evaluate the effect of curcumin on hepatic I/R-induced injury and the underlying mechanism.

Firstly, we investigated the effect of curcumin on hepatic I/R injury conditions *in vitro*. The LDH activity indirectly indicates the degree of cell injury. The major findings of the *in vitro* assessment were that curcumin could suppress LDH release of BRL-3A cells in the culture medium. Additionally, curcumin significantly suppressed TLR4 and NF-κB expression whereas it increased the expression of IκB-α. Hepatic I/R injury can be attributed to apoptosis of liver cells and the infiltration of immune cells. The ELISA data showed that curcumin decreased levels of TNF-α, IL-6, and IL-1β. The flow cytometric analysis also showed that curcumin significantly reduced the apoptosis rates *in vitro*, and. reduced the level of ALT and AST *in vivo*. Moreover, curcumin reduced the production of TNF-α, IL-6, and IL-1β in rat serum. Curcumin showed the inhibition effect as evidenced by the reduction of TLR4 and NF-κB protein expression and elevation of IκB-α expression.

Indicators of hepatocyte injury such as LDH, ALT, and AST, had an important role in the hepatic I/R pathophysiology [14]. ALT is a specific marker for hepatic parenchymal injury and AST is a nonspecific marker for hepatic injury. This study found that serum levels of LDH, ALT and AST were markedly increased. However, curcumin significantly decreased the serum levels of AST, ALT, and LDH, implying that it alleviated hepatic I/R injury.

It is well known that TLR4 plays a role in the activation of inflammatory responses in the setting of acute sterile insults, such as hepatic I/R [15]. TLR4 signaling leads to the phosphorylation of NF-κB and p38 MAPK, which initiate proinflammatory cytokine production [16]. TLR4 blockade could depress the production of proinflammatory cytokines and ameliorates hepatic I/R injury. Previous work has showed that TLR4 is thought to play an important role in the pathogenesis of hepatic I/R injury via the activation of NF-κB signaling [17]. NF-κB, a nuclear transcription factor, plays a critical role in mediating inflammatory and immune responses by involving in the production of both pro-inflammatory cytokines [18–19]. Some pharmacologic agents exert anti-inflammatory and hepatoprotective effects through the TLR4/NF-κB pathway [20–21]. We found that hepatic I/R could upregulate the NF-κB expression and decrease the IκB-α expression. In contrast, curcumin significantly reduced the TLR4 and NF-κB expression while it enhanced the IκB-α expression. In addition, curcumin decreased the production of TNF-α, IL-6, and IL-1β, suggeating that alleviated hepatocyte injury. Together, these findings indicate that curcumin exerts its protective effects by inhibiting the activation of proinflammatory factors *via* the TLR4/ NF-κB signaling pathway.

In summary, our results found that curcumin can confer anti-inflammatory protection via against hepatic I/R injury in the liver, which might have been mediated by the TLR4/NF-κB signaling pathway. Our study suggests that curcumin may be a potential therapy against hepatic I/R injury, and identify TLR4/NF-κB as a major therapeutic target of curcumin action in the liver.

## MATERIALS AND METHODS

### Reagents and drugs

Curcumin was purchased from Sigma-Aldrich (St. Louis, MO, USA). The anti-TLR4 antibody was purchased from Abcam (Product No. ab22048, Cambridge, UK) while the anti-NF-κB and anti-IκB-α antibodies were obtained from Santa Cruz Biotechnology, Inc (anti-NF-κB Product No. SC-372, anti-IκB-α Product No. ZS-371, Santa Cruz, USA). The rabbit anti-goat, goat anti-rabbit and goat anti-mouse secondary antibodies were purchased from Beyotime, China.

### Cell culture

Rat hepatocytes (BRL-3A) were supplied by the Cell Bank of the Type Culture Collection of the Chinese Academy of Sciences (Shanghai, China). They were cultured in Dulbecco’s modified Eagle’s medium (DMEM, Gibco, Grand Island, USA) with 10% (v/v) fetal bovine serum (FBS, Gibco, Grand Island, USA) and antibiotics (100 IU/ml penicillin and 100 mg/ml streptomycin, Gibco, Grand Island, USA) at 37°C in an atmosphere of 95% air/5% CO_2_ in a cell incubator.

### Cell hypoxia/reoxygenation model

Briefly, BRL-3A cells were placed in a tri-gas incubator (Thermo, MA, USA) filled with a mixture of N_2_, CO_2_, and O_2_ (94, 5, and 1%, respectively) for 12 h to achieve the hypoxic condition. Then, the hypoxia-exposed cells were incubated in a normal cell incubator with a mixture of 95% air and 5% CO_2_ for 4 h. There were four experimental groups as follows, normal culture (CON), normal culture with 10 μM curcumin (CON+Cur), and hypoxia/reoxygenation (H/R), as well as hypoxia/reoxygenation with 10 μM curcumin (H/R+Cur) groups. The CON and CON + Cur group cells were cultured in a standard incubator (95% air and 5% CO_2_). The H/R and H/R+Cur group cells all received hypoxia/reoxygenation treatment.

### Animals

Male Sprague-Dawley rats (200–250 g) were purchased from Hua Fukang Experimental Animal Center, Beijing, China, and maintained in a standard environment (natural light/dark cycle, temperature of 23 ± 2°C, and humidity at 60 ± 5%), and given free access to food and water. Animal protocols were approved by the Beijing University Animal Care Committee, and the experiments were performed in adherence to the guidelines provided by the National Institutes of Health for the use of animals in laboratory experiments. After a minimum 7 days of acclimation, the rats were randomly allocated into 3 groups of 6 rats each: (1) the I/R-saline group (I/R), in which the rats were subjected to ischemia for 1 hour; (2) the I/R-curcumin group (Cur), in which the rats were administered curcumin (100 mg/kg) 0.5 hour before I/R induction; and (3) the sham operated group (SHAM).

### Animal model of hepatic I/R

Rats were anesthetized with pentobarbital (40 mg/kg, intraperitoneal). A 3-cm midline incision was performed and the hilum of the liver was exposed. All structures in the portal triad (hepatic artery, portal vein and bile duct) to the left and median liver lobes were occluded using a clip in order to produce 70% hepatic ischemia. The clip was removed after 60 min to allow reperfusion, and the abdomen closed. Sham control rats underwent the same procedure with no vascular occlusion. The rectal temperature was maintained at 37°C throughout the surgery by using a warming pad. Blood and liver samples were collected 6 h after reperfusion and stored at −80°C prior to use.

### Flow cytometry analysis

Flow cytometry analysis was performed to determine whether curcumin could inhibit the growth phase of cells. Cells were seeded into 6-well plates. After treatment with curcumin, BRL-3A cells were collected and resuspended in 500 μl binding buffer and then 5 μl each of Annexin V-FITC, and PI was added to the resuspended cells. The cellular apoptotic rate was evaluated using a FACS VerseTM flow cytometer (Becton Dickinson, CA, USA). Cells for growth phase analysis were resuspended in 200 μl PBS, fixed with 70% ice-cold ethanol overnight, and stained with PI. The cell cycle was detected by the FACSVerse™ flow cytometer.

### Lactate dehydrogenase (LDH) activity assay

The LDH activity wasmeasured in a 20 μl sample of the cell supernatant using an LDH kit (JianCheng Bioengineering Institute, Nanjing, China) using a microplate reader (Biotek, USA).

### Liver damage assessment

To assess hepatic function and cellular injury after liver ischemia, serum aspartate aminotransferase (AST) and alanine aminotransferase (ALT) levels were measured in blood samples obtained at predetermined time points 24 hours after reperfusion by using a standard automatic analyzer (type 7150; Hitachi, Tokyo, Japan).

### Enzyme-linked immunosorbent assay (ELISA)

The levels of the inflammatory cytokines tumor necrosis factor (TNF)-α, interleukin-6 (IL-6), and IL-1β in the culture medium and rat serum were measured using enzyme-linked immunosorbent assay (ELISA) kits (Neobioscience Bioengineering Co., Shenzhen, China) according to the manufacturer›s instructions and the absorbance of the samples were read at 450 nm using a microplate reader (Biotek, USA).

### Real-time polymerase chain reaction analysis

Total RNA was extracted using TRIzol reagent (Invitrogen). Complementary DNA was synthesized using the PrimeScript RT reagent Kit (TaKaRa), according to the manufacturer’s instructions. qRT-PCR was performed using SYBR Green PCR master mix (Applied Biosystems) in a total volume of 10 μl using the Light-Cycler 480 instrument (Roche Diagnostics, Penzberg, Germany) with the following conditions: 95°C for 5 min, followed by 45 cycles of 95°C for 30 sec, 55°C for 30 sec and 72°C for 15 sec, and a final extension step of 72 °C for 5 min. The qRT-PCR results were calculated according to 2^−ΔΔCt^, and all experiments were repeated in triplicate.

### Western blot

Whole-cell lysates were prepared by incubating the cells in radioimmunoprecipitation assay buffer (Beverly, MA) supplemented with a protease inhibitor cocktail (Roche, Mannheim, Germany) according to the manufacturer’s instructions. Briefly, the cells were harvested by centrifugation at 13,200 rpm for 5 minutes and washed in phosphate-buffered saline (pH 7.2). The pellets were solubilized in the same volume of mitochondrial lysis buffer, kept on ice, vortexed for 5 minutes, and then centrifuged at 13,200 rpm for 20 minutes at 4°C. Equal amounts of the total lysate proteins were separated using a 15% SDSePAGE and electrophoretically transferred onto polyvinylidene fluoride membranes, which were blocked with 5% skim milk in Tris-buffered saline and Tween 20 buffer for 1 hour. Then, the membranes were incubated with primary antibodies at 4°C overnight. The membranes were then washed thrice with Tris-buffered saline and Tween 20 buffer and probed with the corresponding horseradish peroxidase-conjugated secondary antibodies at room temperature for 1 hour. The probes were subsequently detected using enhanced chemiluminescence Advance Western Blotting Detection Reagents (GE Healthcare, Buckinghamshire, UK) and EZ-Capture ST imaging system (Atto, Tokyo, Japan).

## IHC

IHC staining for TLR4 was performed on 4 μm thick sections mounted on polylysine-coated glass slides by using the Two-Step IHC Detection Reagent (PV-6001) kit (Zhong Shan Golden Bridge Biological Technology Inc., Beijing, China). The tissue sections, after baked at 60°C for at least 2 h, were deparaffinized with xylene and rehydrated in graded alcohol concentrations and in distilled water. Then, the sections were immersed in 3% hydrogen peroxide for 10 min to inactivate endogenous peroxidase. For antigen retrieval, they were heated in 0.01 mol/l citrate buffer (pH 6.0) in an autoclave at 121°C for 4 min and cooled to room temperature. The sections were incubated overnight with the primary antibody against TLR4 (1: 100, Abcam, Cambridge, MA, USA) at 4°C. After a thorough washing step with PBS, the secondary antibody (Zhong Shan Golden Bridge Biological Technology Inc.) was applied on the sections; and the sections were incubated for 20 min. 3,3′-Diaminobenzidine tetrahydrochloride (Dako, Glostrup, Denmark) was added for visualization. Counterstaining with hematoxylin was performed and the sections were dehydrated in ethanol before mounting.

### Statistical analysis

Student’s *t*-test (two-tailed), One-way ANOVA and Mann–Whitney test were performed to analyze the data using SPSS 16.0 software. *P* values less than 0.05 were considered statistically significant.
